# A low proportion of rare bacterial taxa responds to abiotic changes compared with dominant taxa

**DOI:** 10.1111/1462-2920.14492

**Published:** 2018-12-19

**Authors:** Viola Kurm, Stefan Geisen, Wilhelmina H. Gera Hol

**Affiliations:** ^1^ Department of Terrestrial Ecology Netherlands Institute of Ecology (NIOO‐KNAW) P.O. Box 50 Wageningen 6700 AB The Netherlands; ^2^ Wageningen Plant Research, Biointeractions and Plant Health P.O. Box 16 Wageningen 6700 AA The Netherlands

## Abstract

In many studies, rare bacterial taxa have been found to increase in response to environmental changes. These changes have been proposed to contribute to the insurance of ecosystem functions. However, it has not been systematically tested if rare taxa are more likely to increase in abundance than dominant taxa. Here, we study whether rare soil bacterial taxa are more likely to respond to environmental disturbances and if rare taxa are more opportunistic than dominant taxa. To test this, we applied nine different disturbance treatments to a grassland soil and observed changes in bacterial community composition over 7 days. While 12% of the dominant taxa changed in abundance, only 1% of the rare taxa showed any effect. Rare taxa increased in response to a single disturbance treatment only, while dominant taxa responded to up to five treatments. We conclude that rare taxa are not more likely to contribute to community dynamics after disturbances than dominant taxa. Nevertheless, as rare taxa outnumber abundant taxa with here 230 taxa that changed significantly, the chance is high that some of these rare taxa might act as ecologically important keystone taxa. Therefore, rare and abundant taxa might both contribute to ecosystem insurance.

## Introduction

Since the discovery that most microbial communities are comprised of a large percentage of rare bacterial taxa, also called the ‘rare biosphere’ (Sogin *et al*., 2006), rare taxa have frequently been shown to contribute to a variety of ecosystem functions. While some rare bacterial taxa have been shown to support specific ecosystem processes more strongly than would be expected from their abundance, it has also been demonstrated that many have the ability to become dominant in space and time (Caporaso *et al*., 2012). At increased abundances, these formerly rare taxa have higher functional importance and take over or supplement functions performed by other dominant taxa (Campbell *et al*., 2011; Kearns *et al*., 2017). By increasing in abundance and supporting important functions, rare species are supposed to maintain ecosystem functioning under changes in environmental conditions‐ also called the insurance effect (Yachi and Loreau, 1999). It has been assumed that the high diversity within the rare biosphere increases the probability of including taxa that are adapted to specific abiotic conditions (Flather and Sieg, 2007) and these rare taxa could consequentially support ecosystem functioning after specific environmental changes. Still, previous studies generally tested community changes only in response to a single disturbance treatment or to general seasonal changes (Campbell *et al*., 2011; Ferrenberg *et al*., 2013).

The insurance effect is supposed to be especially strong in diverse communities where community members are adapted to different environmental conditions and thus are habitat specialists (Matias *et al*., 2012). Specialist taxa should increase in abundance in response to particular changes in environmental conditions that benefit them, but not to others. Examples are found in studies that apply specific disturbance treatments to microbial communities. Here disturbances are defined as sudden environmental modifications, including nutrient additions and physical and chemical disturbances. One prominent example of an environmental modification is increased temperature, such as in high temperature composting with temperatures beyond 60 °C that change the bacterial community composition towards a dominance of thermophilic taxa, e.g. bacilli (Liao *et al*., 2018). However, both rare and dominant bacteria can be habitat specialists that respond equally to disturbances (Lindh *et al*., 2015). In addition, current studies examining community changes in response to disturbance did not distinguish between true specialists and taxa that simply benefit from decreased competition as they only assessed one or a small range of disturbances, or a mix of changes such as seasonal dynamics.

Frequently rare taxa have been found to be highly active, as indicated by higher rRNA/rRNA‐gene ratios, rapid changes in abundances and stable isotope probing (SIP) (Campbell *et al*., 2011; Wilhelm *et al*., 2014; Morrissey *et al*., 2016). The high activity of rare taxa indicates that rare taxa are not often dormant or inactive as previously suggested. In addition, dormant cells can be reactivated within few hours after changes in the environment and are therefore part of the extended active bacterial community (Aanderud *et al*., 2015). Taxa fluctuating between states of dominance and rarity have been termed conditionally rare taxa (Shade *et al*., 2014). Similarly, using deep sequencing in seawater collected over multiple seasons, Caporaso and colleagues (2012) observed that the vast majority of taxa that gained temporal dominance were always present during the year. Abundance of these taxa fluctuated between being dominant and rare, but they were permanent members of the community. While high activity might enable rapid increases of rare bacterial taxa in response to environmental changes, it has not been tested yet whether rare taxa are more likely to increase in abundance or whether this observation is a consequence of the overall high number of rare taxa.

In addition to their high activity under the right conditions, conditionally rare taxa are hypothesized to be on the more copiotrophic side of the spectrum from oligotrophic to copiotrophic lifestyles (Newton and Shade, 2016). Copiotrophic bacteria have been defined as those that exhibit fast growth under high nutrient concentrations, as opposed to oligotrophic bacteria that have rather slow growth rates but are able to thrive under extremely low nutrient concentrations (Fierer *et al*., 2007). In addition, fast growth has frequently been found to be associated with a diminished competitive ability (Kurihara *et al*., 1990; Harpole and Tilman, 2006; Burton *et al*., 2010). This trade‐off could lead to a low abundance of fast growing copiotrophic taxa under stable conditions, whereas competitively superior taxa become dominant. In response to disturbance regimes and environmental changes, which cause dominant, competitive taxa to decline, rare fast‐growing taxa might increase. However, this can only occur if they are also generalists that are able to cope with a variety of environmental conditions. Here we define taxa that are both fast growing and habitat generalists as opportunistic. The increase of many rare taxa in response to environmental changes indicates that these taxa might not only be copiotrophs but also opportunistic.

Different theories have been proposed to explain why some taxa are rare, whereas others are dominant, such as the R* theory (Tilman, 1982) stating that the species with the capability to reduce a limiting resource to the lowest level (i.e. has the lowest R*) should outcompete all other species. Harpole and Tilman (2006) could show that species with a lower R* for N were more competitive and thus more abundant than species with a higher R*. In addition, different concentrations of the resource changed species relative abundance. In the study by Harpole and Tilman (2006) competition between taxa at different resource‐availabilities was found to determine taxon abundance. However, current knowledge on the influence of R* and competition on abundance is derived from studies on plants and it is currently unknown to what extent the R* theory is applicable to bacterial communities. The C,‐S,‐R‐theory by Grime (1977), on the other hand, proposes differing strategies of organisms, which consequentially will have a competitive advantage under different environmental conditions. This is in accordance with numerous studies observing trade‐offs between e.g. traits that increase competition, such as using a limiting resource, and traits increasing tolerance to certain stresses (Kneitel and Chase, 2004). Such trade‐offs have been found to exist among multiple traits and environmental conditions. Thus, an organism's abundance is an outcome of its balance of strategies and the current environmental conditions. For example, stress tolerant taxa might be rare in stable environments, but could be selected for under disturbance treatments.

Consequently, rare taxa that have the ability to rapidly become dominant due to their ability for fast growth and opportunistic lifestyle could be expected to decrease in abundance after a disturbance when competitively superior taxa recover. This is in line with studies reporting fluctuating taxon abundances and several blooms and subsequent crashes of populations of rare taxa with returning seasonal changes (Shade *et al*., 2014). Similarly, taxa that are not opportunistic, but adapted to a specific disturbance or environmental change are likely to show decreases in abundance as the community recovers from the disturbance (Jurburg *et al*., 2017). However, this recovery of the community should only occur in response to single short‐term disturbances (pulse‐disturbances). Long‐term changes in environmental conditions (press‐disturbances) are more likely to permanently alter microbial community composition (Shade *et al*., 2012) and specialist taxa that are adapted to certain conditions might become permanently dominant if the environment remains favourable. Opportunistic taxa, on the other hand, should decline in abundance after pulse‐ as well as press‐disturbances.

The increase in abundance of rare taxa in response to environmental changes has been observed frequently and is supposed to be an important mechanism underlying the insurance effect (Szabo *et al*., 2007). However, it is still unclear if changes in abundances differ between rare and dominant taxa. The distinction between rare and dominant taxa and their response to environmental change can be of particular importance when attempting to predict changes in community composition and functioning. In this study, we investigate whether rare bacterial taxa behave as opportunists upon disturbances by testing the following hypothesis: (i) Rare bacterial taxa are more likely to exhibit changes in abundance in response to disturbance treatments than dominant taxa; (ii) Rare bacterial taxa increase in abundance in response to more than one disturbance treatment; (iii) Rare taxa will rapidly increase in abundance and then decrease again after a disturbance event (i.e. are more likely to fluctuate).

To test these hypotheses, we applied 9 different disturbances, which we here define as sudden or constant changes in the environment, to soil microcosms and compared the bacterial community composition with the control treatment. Seven treatments were pulse disturbances (wheat‐straw addition, glucose‐amendment, addition of nitrogen, addition of copper, heat shock, freeze thawing and microwave treatment), whereas two were press disturbances (mechanical disturbance and drying and rewetting). Soil samples were sequenced by 454‐ pyrosequencing to assess relative changes in bacterial taxon abundances at day 1, 3 and 7 after disturbances.

## Results

**Figure 1:**
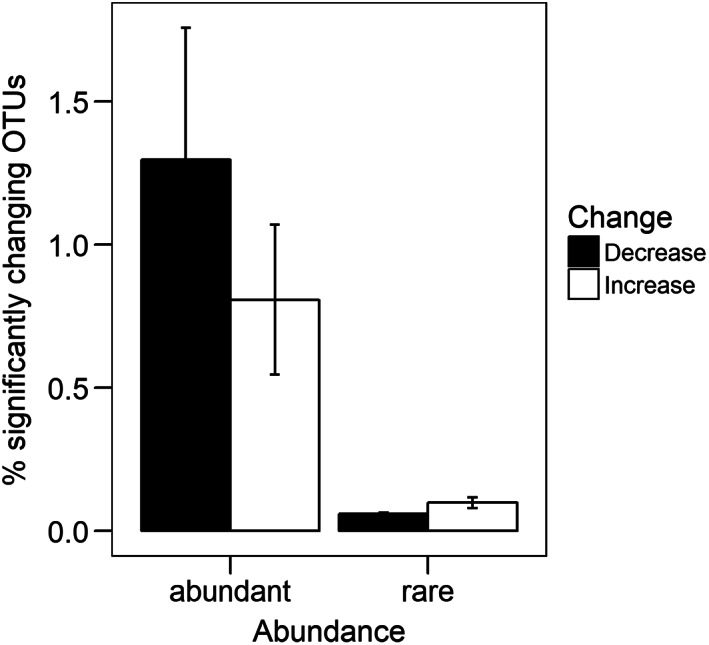
Percentage of dominant and rare OTUs respectively that change a) in response to disturbance treatments, either increasing or decreasing in abundance (*p* < 0.05); error bars represent the standard error; data represents averages over the disturbances.

Of all 20 325 operational taxonomic units (OTUs) that we conservatively defined based on 97% sequence similarity and consequently defined as taxa, 94% were classified as rare (<0.01% relative abundance of all bacterial 16S rRNA gene reads) and 6% as dominant (>0.01%). Note, that all sequence data is expressed as relative abundances as inherent to high‐throughput sequencing approaches. Disturbances caused significant changes of relative abundance in 1.6% of bacterial taxa in all treatments combined. Dominant taxa were more likely to change in abundance in response to disturbance treatments than rare taxa (*t* = −3, *p* = 0.02; Fig. 1) compared with the untreated control.

Of all rare taxa 1% significantly changed in abundance in the disturbance treatments with 0.9% increasing and 0.09% decreasing. Of all dominant taxa 12% significantly changed in abundance with 6.2% increasing and 5.8% decreasing.

**Figure 2:**
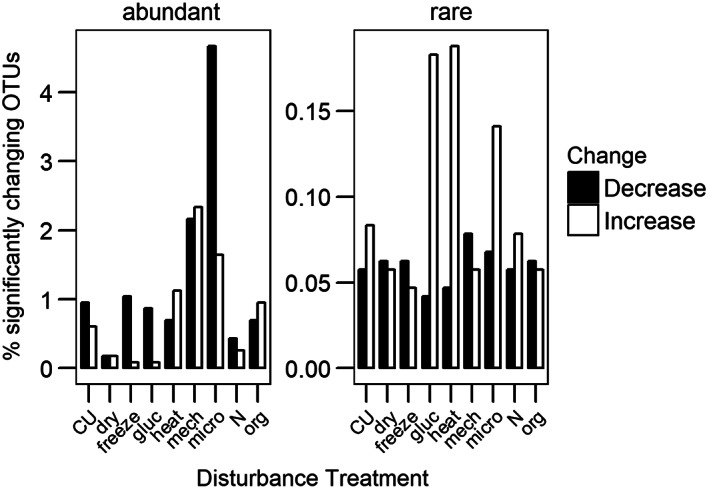
Average relative abundance of significantly decreasing and increasing dominant and rare OTUs in the nine disturbance treatments.

Rare taxa attained on average highest abundances in the heat treatment with an average relative abundance of 0.2% (*t* = 5.5, *p* < 0.01; Fig. 2). Six taxa classified as rare reached abundances above 1% in the heat treatment. All were identified as belonging to the order Bacillales.

### *Changes in taxon relative abundances due to different disturbances*

**Figure 3:**
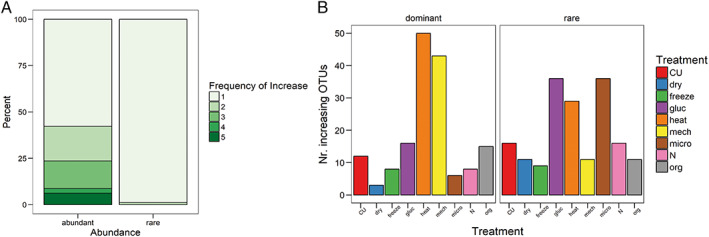
A. Percentage of dominant and rare OTUs increasing significantly in response to one or more disturbance treatments (*p* < 0.05); B, number of dominant and rare OTUs increasing significantly in response to the nine disturbance treatments.

Of all significantly increasing taxa that were classified as rare the majority (98.9%) changed in response to only one type of disturbance and 1.1% changed in response to two types of disturbances (Fig. 3A). Most taxa that were classified as rare increased in response to glucose, heat and microwave disturbances (Fig. 3B). Similarly, significantly increasing taxa that were classified as dominant (57.8%) increased most in response to 1 type of disturbance, but a larger fraction of the remaining taxa simultaneously increased in response to two to five types of disturbance (Fig. 3A). However, there was no significant difference between rare and dominant taxa with respect to the number of disturbances they responded to.

Most dominant taxa increased in response to heat and mechanical disturbance (Fig. 3B).

### *Dynamics of abundance increase*

**Figure 4:**
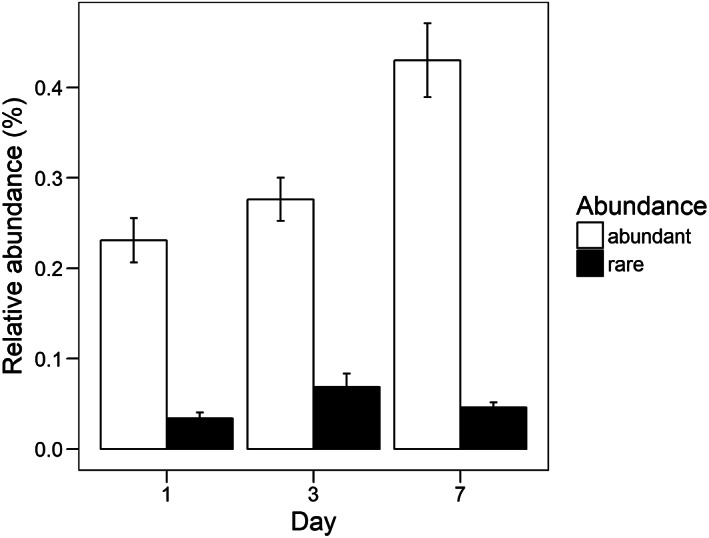
Average relative abundance of significantly increasing OTUs across all treatments that were classified as rare or dominant on the three different sampling days; error bars indicate the standard error; significant differences between days within the two groups are indicated by different letters.

**Figure 5:**
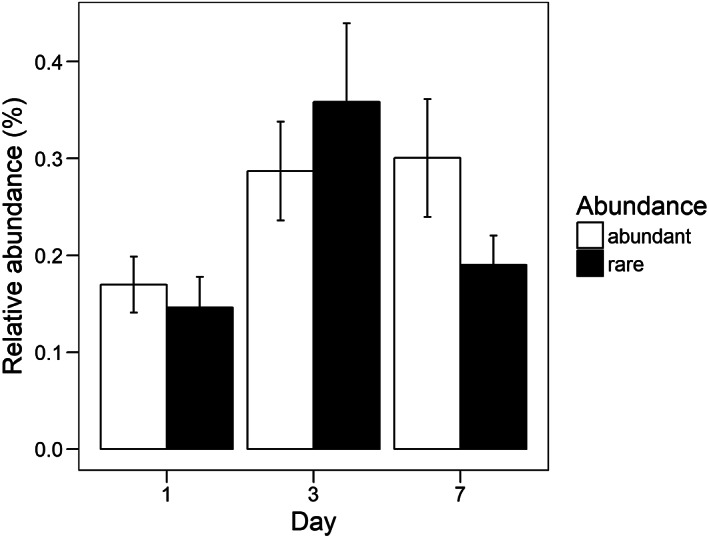
Average relative abundance of significantly increasing OTUs classified as rare or dominant on the three different sampling days with the heat treatment; error bars indicate the standard error; significant differences between days within the two groups are indicated by different letters.

Averaged over all treatments there was no difference in the relative abundance of significantly increasing rare taxa between day 1, 3 and 7. In contrast, dominant taxa increased significantly from day 1 to day 7 (day 1–day 3: *t* = 2.7, *p* < 0.01, day 1–day 7: *t* = 8.5, *p* < 0.01, day 3–day 7: *t* = 5.7, *p* < 0.01; Fig. 4). We found a marginally significant interaction effect between abundance group, day of sampling and disturbance treatment (*F* = 1.7_16_, *p* = 0.05). The relative abundance of rare taxa in the heat treatment increased significantly from day 1 to day 3 and decreased again at day 7 (day 1–day 3: *t* = −3.9_1340_, *p* < 0.01, day 3–day 7: *t* = 3.1_1340_, *p* < 0.01), while for dominant taxa the relative abundance increased significantly from day 1 to day 3 and 7 (day 1–day 3: *t* = −2.8_1340_, *p* < 0.01, day 1–day 7: *t* = −3.1_1340_, *p* < 0.01; Fig. 5). For dominant taxa, there were additional differences in relative abundances between the different days for the mechanical, microwave, glucose and copper sulfate disturbances but not for the other disturbances. Relative abundance increased from day 1 to day 7 in the mechanical, microwave and copper sulfate disturbance. For the glucose disturbance relative abundance was highest on day 3 (Supporting Information Fig. S1). There was no difference in the dynamics of abundance changes for rare taxa in response to pulse disturbances compared with press disturbances (rare: *F* = 2.5_2724_, *p* = 0.09). For dominant taxa there was a stronger increase in abundance with press disturbances compared with pulse disturbances (*F* = 17.3_2675_, *p* < 0.01).

## Discussion

In contrast to our expectations, only a small percentage of rare soil bacterial taxa significantly increased in abundance in response to the various disturbance treatments and rare taxa were less likely to increase than dominant taxa. Although the total number of rare taxa responding is higher with 230 taxa than that of the dominant taxa, this can be attributed to the high general number of rare taxa, increasing the chance of containing taxa that will respond. Therefore, our results do not support our first hypothesis that rare taxa are more likely to increase in abundance in response to disturbances. Our results indicate that rare taxa are neither more likely to be opportunistic and able to quickly exploit the decline of dominant taxa nor do they contain relatively more specialists that are adapted to specific changes. While several rare taxa belonging to the order Bacillales became dominant in response to the heat treatment, also dominant taxa of the Bacillales increased in abundance. This is in agreement with a general increase of taxa within Bacillales as during high temperature composting (Liao *et al*., 2018). This indicates that rare as well as dominant taxa have the ability to tolerate specific disturbances and is consistent with a study by Low‐Décarie and colleagues (2016) finding no trade‐off between the ability to grow under extreme and under benign conditions.

The weak response of rare taxa is surprising since former studies routinely reported large parts of the rare biosphere to fluctuate in abundance over time, particularly after changes in the environment (Campbell *et al*., 2011; Shade *et al*., 2014). This could be due both to our conservative threshold of 0.01% for the definition of rarity and to the stringent procedure for determining significant changes in abundances. The formerly often used threshold of 1% to define rare taxa (Campbell *et al*., 2011; Shade and Gilbert, 2015) would have increased the number of taxa to be recruited from the rare biosphere leading to a proportion similar to those found in previous studies (Campbell *et al*., 2011; Shade *et al*., 2014). However, with the technological advances that enable deeper community analysis we believe that the rare biosphere can be analysed more thoroughly and propose a threshold <0.01% for rare bacterial taxa. Previous studies have mostly described the number of (rare) taxa that change in abundance in response to disturbance treatments, but here we assessed the significance and variability of this response.

We also observed that rare taxa on average increased in response to only a single type of disturbance treatment, while some dominant taxa responded to up to five types of disturbances. These results did not support our second hypothesis that rare bacterial taxa would increase in abundance in response to several types of disturbances. Still, rare and dominant taxa did not significantly differ with respect to the number of treatments that led to an increase in abundance. Together with the observation that rare taxa are not more likely to increase in abundance this finding supports the conclusion that rare taxa are not more opportunistic than dominant taxa. The response to only a single treatment indicates that most taxa that do increase possess specific response traits that enable them to increase in abundance in response to a specific set of conditions that favour their growth as has been suggested previously (Flather and Sieg, 2007). Still, rare taxa cannot be considered to be more likely to be habitat specialists than dominant taxa as they were overall less likely to respond to disturbances.

We also did not observe that rare taxa, which increase in abundance in response to a disturbance, subsequently decline again. Therefore, we reject our third hypothesis. The lack of a decline can be due to the low number of taxa that initially increased in abundance. However, even taxa that increased in abundance did not show fluctuations over the sampling days, regardless if they were subjected to pulse‐ or press‐disturbances. This indicates that rare taxa might not be opportunistic and therefore subject to sudden blooms and subsequent decline as has been suggested in previous studies (Newton and Shade, 2016). However, the time period of observation might have been too short to detect such a decline. A study by Jurburg and colleagues (2017) demonstrated that, in response to heat, the decrease in abundance of initially as well as of later responding taxa can take place between 10 and 30 days after disturbance. In the present study, we also found that dominant bacterial taxa that increase in abundance do not decrease again, but continue to increase until day 7 supporting the assumption that a too short observation time prevented us from detecting a decline. Only in the heat treatment an initial increase and subsequent decrease of the abundance of rare bacteria could be observed, but not of dominant bacterial taxa that were still increasing at the latest sampling point. This difference suggests that these heat‐responding rare bacterial taxa might be true specialists, but weak competitors and therefore quickly decline when other taxa continue to grow or recover from disturbance. In addition, this study consisted of a limited range of disturbance treatments. Other environmental changes and especially seasonal dynamics that are involved in the alteration of several abiotic factors, might affect rare taxa differently.

The rejection of all three hypothesis indicates that rare taxa are not more likely to be opportunistic than dominant taxa. Although it has been demonstrated that some have the ability to grow rapidly (Baldrian *et al*., 2012; DeAngelis and Firestone, 2012), we do not find evidence that the rare biosphere on average contains a higher proportion of taxa that are able to exploit the decline of potential competitors. Although many dominant taxa decreased in abundance in the disturbance treatments, only few rare taxa significantly increased. Our sequence‐based analysis focused on DNA and might also detect taxa that are already dead or inactive, especially at the first sampling (Carini *et al*., 2016). This could lead to an underestimation of the percentage of changing taxa, but as DNA degrades at a constant rate and was found to not affect the outcome of bacterial community analyses, our results are likely not impacted by DNA originating from dead taxa. Our results suggest that disturbances lead to a turnover of dominant taxa containing both opportunistic taxa and those which profit from specific changes, while most rare taxa stay permanently rare. Still, the overall high number of rare taxa increases the probability of the community containing some taxa that become dominant in response to one of the disturbances (Fetzer *et al*., 2015; Low‐Décarie *et al*., 2015). Thus, the rare biosphere as a whole is still likely to play an important role in ecosystem insurance. However, in this study we did not assess ecosystem functions and therefore cannot speculate about the community resistance or resilience with respect to functioning.

The lack of a response of most rare taxa indicates that many might be consistently rare, which has also been reported in some studies (Gobet *et al*., 2012), but not in others (Campbell *et al*., 2011). These permanently rare taxa might be dormant or extremely slow growing (Pedros‐Alio, 2007), while other factors that were not altered by disturbance might constrain their abundance. For example, rare bacterial taxa might be reduced to low abundances by grazing as soil harbours a wide variety of predators, such as protists and nematodes (Ekelund and Rønn, 1994). The kill‐the‐winner hypothesis by Thingstad and Lignell (1997) suggests that fast growing and highly competitive taxa are more strongly reduced by predation, which could explain low abundances of active taxa. This is supported by a study from Neuenschwander and colleagues (2015) who found rare bacterial taxa with the potential for fast growth to be tightly controlled by predation. However, the causes for permanent or temporal rarity are still poorly studied.

We conclude that over a short period of time a higher proportion of dominant than of rare bacterial taxa changes in abundance in response to environmental disturbances. It remains to be studied if rare taxa will show stronger increases at a later time point after disturbances as studied here. Still, the high number of rare taxa increases the probability of the rare biosphere to contain taxa that are tolerant to a wide range of disturbances, which can play important roles in ecosystem functioning after environmental changes.

## Experimental procedures

### *Soil collection and sample processing*

Soil was collected on February 18, 2013 from a plot (HD5) with high plant diversity from all functional groups (grasses, forbs, legumes) from a long‐term diversity experiment ‘CLUE’ in Ede, the Netherlands (52° 3′ 35.95 N 5° 45′ 10.63E). The field is well studied for a variety of abiotic and biotic measurements, followed since abandoning agricultural management in 1995 (Van der Putten *et al*., 2000). Samples were collected from three different locations on a linear transect within the plot, with ~ 2 m between closest locations. For each location, three samples were collected from a triangular grid with a soil gouge (3 cm ø, 30 cm deep) with ~ 30 cm between samples. The choice of the sampling within a plot as opposed to more diverse and spatially separate sites was based on the desire to get a good estimate of the locally rare bacteria. For each sample, one Eppendorf tube was filled in the field with ~1 g soil from 10 cm depth and stored on ice for 1 h until arrival at the institute where it was frozen at −20 °C (3 replicates, 3 locations = 9 samples). The remaining soil was sieved to remove stones and roots, and pooled per sampling location. Two Eppendorf tube was were filled with ~1 g pooled soil for each of the 3 soil origins and stored at −20 °C for one week until DNA extraction.

### *Disturbance treatments*

For each of the three soil samples, 20 100 ml autoclaved Erlenmeyers were filled with 15 g fresh weight soil. The soils were exposed to several abiotic disturbances. There were 10 treatments in total that are detailed in Table 1, and two technical replicates for each soil sample. The technical replicates were more similar to each other than to the other sampling locations (Supporting Information Fig. S2). The 10 treatments were: addition of wheat straw, addition of glucose, addition of copper sulfate, addition of nitrogen, heat shock, freezing–thawing, mechanical disturbance, microwave treatment and moisture fluctuations (dry‐rewet). The additions were all mixed as dry compounds with the soil, while the other treatments received a similar homogenization. While not all disturbance treatments link to ecological relevant conditions, they do simulate a large variety of different potential stressors. Most treatments were applied only once, except for the mechanical disturbance and drying‐rewetting. The mechanical disturbance consisted of daily stirring with a magnetic stirrer, resulting in compacted soil. The dry‐rewet treatment was evaporating for 16 h day^−1^ in a laminar flow cabinet, with daily adjustment of the moisture to starting level. The Erlenmeyers were stored in the dark at 15 °C, representing average soil temperature in the growing season and covered with aluminium foil. Weight was adjusted daily to the starting weight; on average 0.35 ml for the drying‐rewetting treatment and only 0.02 ml for the other treatments. Soil samples were taken at several time points (1, 3, 7 days after disturbance) to assess the community structure and relative abundances of bacterial taxa (10 disturbances × 3 time points × 3 soils × 2 technical replicates = 180 samples).

**Table 1:** Disturbance treatments applied to each 15 g soil sample with two technical replicates.

Abbreviation	Name	Description
Org	Organic material	Mixing of 30 mg finely ground dried leaves from *Triticum aestivum* with soil
Gluc	Glucose	Mixing of 30 mg glucose with soil
CU	Copper	Addition of 24.45 mg CuSO_4_
N	Nitrogen	Addition of 7.2 mg NH_4_NO_3_
Heat	Heat shock	60 min at 60 °C in a hot air oven
freeze	Freezing	24 h at −18 °C in a freezer
mech	Mechanical	Daily 10 min with magnetic stirrer resulting in increasing compaction
micro	Microwave	Daewoo KOR 6105; 385 W for 30 s
dry	Dry rewet	Evaporation for 16 h day^−1^; moisture adjusted daily to starting moisture
CTRL	Control	No addition

### *Molecular analysis of microbial community composition*

Bacterial community composition in the soil samples was assessed via partial 16S rRNA gene sequencing, as described in Hol and colleagues (2015). Briefly, DNA extraction was performed on 0.25 g soil with the Mobio Powersoil© DNA isolation kit according to the manufacturer's protocol. DNA concentrations were estimated with Nanodrop. DNA was amplified in a 50 μl PCR reaction in a C1000 Touch™ Thermal Cycler with primers targeting the V4 region (515f, 806R; Lauber and colleagues (2009)). Samples were purified after PCR with the Qiagen DNA purification kit. Sequencing of the 16S rRNA gene was done on 454 FLX platform with Macrogen (South Korea). The nine field samples and three sieved samples were placed on half a plate, to get sufficient coverage of the rare biosphere. The 180 samples of the 1, 3, 7 days after treatment were randomly distributed over 1.5 plates. Sequence analysis was done as described in Navarrete and colleagues (2013) and Hol and colleagues (2015). In short, we kept only high quality sequences with perfect matches in the primer and barcode region and that contained no homopolymers above six nucleotides. Denoiser 0.91 (Reeder and Knight, 2010) and UCHIME version 4.2.40 (Edgar *et al*., 2011) were used for denoising and chimeral removal. Remaining sequences in the range of 300–380 bp were kept and trimmed to bases with quality scores only higher than 25. This resulted in a total of 1 216 172 reads with 20 000–50 000 reads for the field soil samples and an average of 6600 per sample from the disturbance treatments. UCLUST version 1.2.21 (Edgar, 2010) was used to cluster these reads into Operational Taxonomic Units (OTUs) of 97% similarity resulting in 20 325 OTUs, here defined as taxa. These taxa were taxonomically assigned using the Ribosomal Database Project (RDP) 2 classifier (release 10.4), with a minimum support threshold of 60%.

### *Data analysis*

All analysis was conducted in R Studio (R Core Team, 2016).

In order to test which taxa changed significantly in relative abundance in response to a disturbance treatment pairwise *T*‐tests were conducted between relative abundances in the disturbance treatments and in the control for each taxon in all three samples using the pairwise.t.test() function with *p*‐value adjustment according to the Holm method (Holm, 1979). Subsequently, the proportion of significantly changing taxa that were either rare (relative abundance <0.01% or dominant >0.01%) in the control was calculated for each treatment and a *T*‐test was conducted between the proportions of changing rare and dominant taxa across all treatments to enable to statistically analyse differences within and between treatments. To assess, if there are differences between the frequencies of increase in abundance between rare and dominant taxa the proportion of taxa that increased significantly in response to 1–9 disturbances was calculated and a chi‐square test was conducted comparing how many taxa increased significantly in response to how many treatments (min = 1, max = 7).

To assess whether taxa that increase in abundance in response to a disturbance treatment also rapidly decline again and whether this response differs between rare and dominant taxa a linear mixed model was used with the lmer() function from the lme4 package (Bates *et al*., 2015). The dataset used contained only taxa that have been found to increase significantly in abundance. Relative abundance was used as the response variable and abundance category (rare or dominant) and day of sampling (day 1, day 3, day 7) were the explanatory variables. Taxon identity and sequencing plate were random variables. The same test was conducted with a subset of significantly increasing taxa for each treatment. Subsequently the minimal model and pairwise differences between the factor levels were determined using the step() function from the lmerTest package (Barberan *et al*., 2012).

## Supporting information


**Fig. S1.** Average relative abundance of significantly increasing OTUs classified as rare or abundant on the three different sampling days and with the different disturbance treatments; errorbars represent the standard error. Abundant OTUs changed significantly in abundance over the sampling days in the heat treatment (*F* = 11.4, *p* < 0.01), in the mechanical disturbance treatment (*F* = 6–1, *p* < 0.01), in the microwave treatment (*F* = 16.6, *p* < 0.01), in the glucose treatment (*F* = 5.7, *p* < 0.01) and in the CU treatment (*F* = 9.8, *p* < 0.01).
**Fig. S2.** Relative abundance of the five most dominant OTUs in the freezer treatment at day 1. Example of the variation within (technical replicates) and between (locations).
OTU1: Proteobacteria Alphaproteobacteria Rhizobiales Bradyrhizobiaceae Bradyrhizobium; OTU2: Chloroflexi KD4‐96
OTU3: Verrucomicrobia Spartobacteria Chthoniobacterales DA101_soil_group
OTU4: Bacteria Actinobacteria Actinobacteria Micrococcales Micrococcaceae Arthrobacter
OTU5: Bacteria Verrucomicrobia Spartobacteria Chthoniobacterales DA101_soil_group
