# Supplementary material for: A low proportion of rare bacterial taxa responds to abiotic changes compared with dominant taxa
Source: Environ Microbiol. 2018 Dec 19;21(2):750–8. doi: 10.1111/1462-2920.14492 (PMC7379498; doi:10.1111/1462-2920.14492)
Supplement: Supplementary file 1 — Fig. S1. Average relative abundance of significantly increasing OTUs classified as rare or abundant on the three different sampling days and with the different disturbance treatments; errorbars represent the standard error. Abundant OTUs changed significantly in abundance over the sampling days in the heat treatment (F = 11.4, p < 0.01), in the mechanical disturbance treatment (F = 6–1, p < 0.01), in the microwave treatment (F = 16.6, p < 0.01), in the glucose treatment (F = 5.7, p < 0.01) and in the CU treatment (F = 9.8, p < 0.01). Fig. S2. Relative abundance of the five most dominant OTUs in the freezer treatment at day 1. Example of the variation within (technical replicates) and between (locations). OTU1: Proteobacteria Alphaproteobacteria Rhizobiales Bradyrhizobiaceae Bradyrhizobium; OTU2: Chloroflexi KD4‐96 OTU3: Verrucomicrobia Spartobacteria Chthoniobacterales DA101_soil_group OTU4: Bacteria Actinobacteria Actinobacteria Micrococcales Micrococcaceae Arthrobacter OTU5: Bacteria Verrucomicrobia Spartobacteria Chthoniobacterales DA101_soil_group [file EMI-21-750-s001.docx]

**Supplementary information**


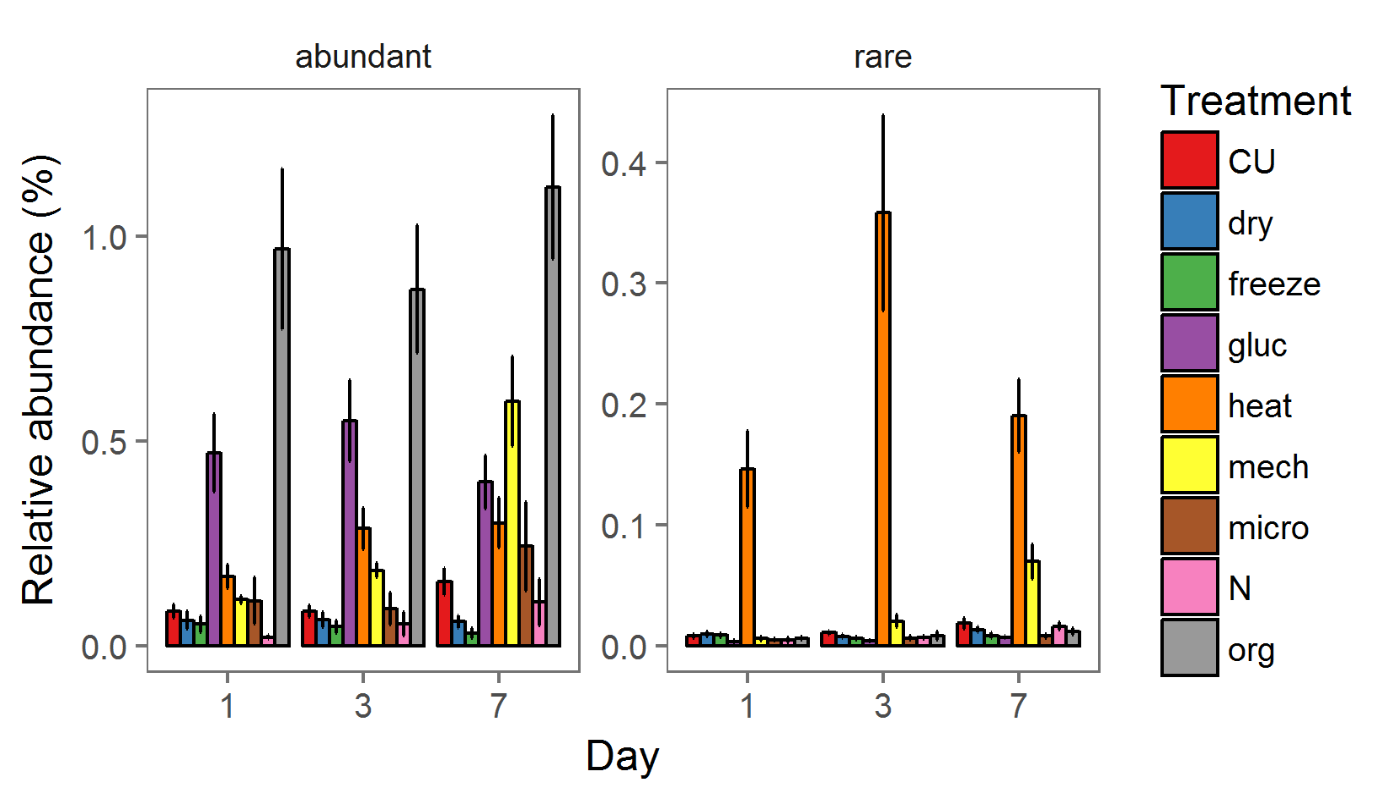


Fig. S1: Average relative abundance of significantly increasing OTUs classified as rare or abundant on the three different sampling days and with the different disturbance treatments; errorbars represent the standard error. Abundant OTUs changed significantly in abundance over the sampling days in the heat treatment (F=11.4, p<0.01), in the mechanical disturbance treatment (F=6-1, p<0.01), in the microwave treatment (F=16.6, p<0.01), in the glucose treatment (F=5.7, p<0.01) and in the CU treatment (F=9.8, p<0.01).


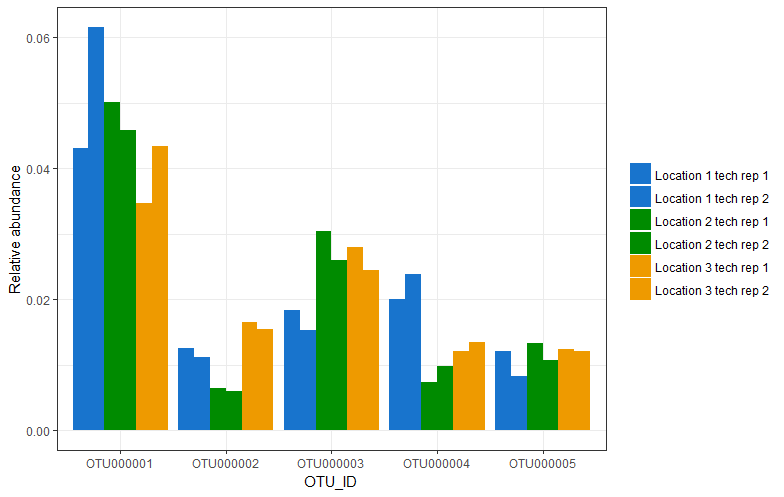


Fig S2: Relative abundance of the five most dominant OTUs in the freezer treatment at day 1. Example of the variation within (technical replicates) and between (locations).

OTU1: Proteobacteria Alphaproteobacteria Rhizobiales Bradyrhizobiaceae Bradyrhizobium; OTU2: Chloroflexi KD4-96

OTU3: Verrucomicrobia Spartobacteria Chthoniobacterales DA101_soil_group

OTU4: Bacteria Actinobacteria Actinobacteria Micrococcales Micrococcaceae Arthrobacter

OTU5: Bacteria Verrucomicrobia Spartobacteria Chthoniobacterales DA101_soil_group
